# PReS-FINAL-2254: Is it systemic JIA or post infectious illness?

**DOI:** 10.1186/1546-0096-11-S2-P244

**Published:** 2013-12-05

**Authors:** P Guddeti, Z Ahmed, M Yiallourides

**Affiliations:** 1 Department of Paediatrics, Pilgrim Hospital, Boston, Lincolnshire, UK; 2 Paediatric Rheumatology, Nottingham University Hospitals, Nottingham, UK

## Introduction

This is a case report of a 15 year old girl which presented with Pyrexia of Unknown Origin. She was initially treated for possible sepsis and then received treatment for Atypical Kawasaki's. She was subsequently investigated for Haemophagocytic Lymphohistiocytosis (HLH). She was eventually diagnosed with Systemic Juvenile Idiopathic Arthritis (SoJIA) complicated with Macrophage Activation Syndrome (MAS) at presentation and was commenced on Methotrexate.

## Objectives

This case report highlights the difficulties in distinguishing inflammatory conditions at first presentation.

## Methods

The case was derived from a retrospective review of case notes and laboratory findings. The patient was managed at two sites, a district hospital paediatric unit and a tertiary rheumatology centre. All authors have been involved in the medical management of the patient.

## Results

A previously well 15 year-old girl presented with two week history of fever, rash, poor appetite, weight loss and multiple joint pain and swelling. She was found to have a non-specific rash, migratory synovitis, muscle weakness, cervical and axillary lymphadenopathy and splenomegaly. Blood investigations had shown anaemia, neutrophilia, thrombocytosis, raised inflammatory markers (CRP, ESR, Ferritin), and deranged liver and muscle enzymes. She was treated initially with broad spectrum IV antibiotics. Infection screen was negative as well as serology for ASOT, anti-Dnase B Streptococcal, EBV, CMV and HIV. In view of persistent pyrexia, lymphadenopathy and rash a diagnosis of Atypical Kawasaki's was made and IV Immunoglobulins were given. Baseline Echocardiogram was normal, abdominal ultrasound confirmed splenomegaly and CT showed evidence of bilateral pleural effusions and a large axillary lymph node.

In view of on-going symptoms she was transferred to a Tertiary unit for further investigations. A diagnosis of Secondary HLH was considered in view of on-going fever, rash, lymphadenopathy, splenomegaly, very high ferritin (160,000), high triglycerides and high SCL25(IL-2R). A bone marrow was inconclusive and no Perforin mutations were identified. She was treated with high dose anti-inflammatories and by the 6^th ^week of her illness her systemic symptoms and rash have subsided however she continued to have persistent synovitis and splenomegaly. A diagnosis of SoJIA was made and was treated with two IV Methyl Prednisolone pulses, intra-articular steroids and commenced on Methotrexate. Following six weeks of Methotrexate all her symptoms had resided and all inflammatory markers were back to normal. As she was intolerant to Methotrexate it was decided to stop treatment. She has remained well since.

## Conclusion

Secondary HLH can be trigger by infections usually EBV, CMV, parvovirus, herpes simplex, varicella-zoster, measles as well as human herpes virus-8 and HIV infection. In our case no infection trigger was identified however our patient met the defined set of Diagnostic criteria^1^. In a new presentation, it is often difficult to make a distinction between infection triggered HLH and a new presentation of systemic JIA complicated with MAS. Early recognition is important and treatment should be initiated promptly.

## Disclosure of interest

None declared.
